# The Associations of Single Nucleotide Polymorphisms of the COL3A1, COL6A5, and COL8A1 Genes with Atopic Dermatitis

**DOI:** 10.3390/jpm13040661

**Published:** 2023-04-13

**Authors:** Krzysztof Szalus, Weronika Zysk, Jolanta Gleń, Monika Zabłotna, Roman J. Nowicki, Magdalena Trzeciak

**Affiliations:** 1 Department of Dermatology, Venereology and Allergology, Faculty of Medicine, Medical University of Gdansk, 80-214 Gdańsk, Poland; kszalus@gumed.edu.pl (K.S.); jglen@gumed.edu.pl (J.G.); monika.zablotna@gumed.edu.pl (M.Z.); roman.nowicki@gumed.edu.pl (R.J.N.); 2 Dermatological Students Scientific Association, Department of Dermatology, Venereology and Allergology, Faculty of Medicine, Medical University of Gdansk, 80-214 Gdańsk, Poland; weronikazysk@gumed.edu.pl

**Keywords:** atopic dermatitis, single nucleotide polymorphisms, type III collagen, type VI collagen

## Abstract

The pathophysiology of atopic dermatitis (AD) is complex, multifactorial, and not fully understood. Genes encoding collagens, the most abundant proteins in the extracellular matrix (ECM), may play a potential role in the pathogenesis of AD. Our study aimed to estimate the associations between *Col3A1*/rs1800255, *Col6A5* /29rs12488457, and *Col8A1*/rs13081855 polymorphisms and the occurrence, course, and features of AD in the Polish population. Blood samples were collected from 157 patients with AD and 111 healthy volunteers. The genotype distribution of the investigated collagens genes did not differ significantly between the AD and control subjects (*p* > 0.05). The AA genotype of *Col3A1*/rs1800255 was significantly associated with the occurrence of mild SCORAD (OR = 0.16; 95% Cl: 0.03–0.78; *p* = 0.02) and mild pruritus (OR = 18.5; 95% Cl: 3.48–98.40; *p* = 0.0006), while the GG genotype was significantly associated with severe SCORAD (OR = 6.6; 95% Cl: 1.23–32.35; *p* = 0.03). Regarding *Col6A5*/29rs12488457 polymorphism, the average SCORAD score was significantly lower in the group of patients with genotype AA than in patients with the AC genotype (39.8 vs. 53.4; *p* = 0.04). Nevertheless, both average SCORAD scores were high, and represent the moderate and severe grades of the diseases, respectively. The single nucleotide polymorphisms (SNPs) of *COL3A1*/ rs1800255 and *Col6A5*/29rs12488457 seem to be associated with AD courses and symptoms, suggesting new disease biomarkers. The modulation of collagens, the major component of the ECM, may serve as a therapeutic target of AD in the future.

## 1. Introduction

Atopic dermatitis (AD) is a recurrent, chronic, inflammatory, and itchy dermatosis; it mainly affects the pediatric population with a frequency of up to 20%, but also occurs in up to 3% of adults [1]. The pathophysiology of the disease is complex, multifactorial, and not fully understood. It consists of epidermal barrier defects, genetic disorders, altered immune response, IgE-mediated hypersensitivity, and environmental factors [2]. The most important genes involved in AD pathogenesis are genes encoding structural and functional proteins of the epidermis, and genes encoding proteins that regulate the innate and acquired immune response. The best-known genetic defect associated with AD is the filaggrin gene mutation leading to an impaired skin barrier, which is a hallmark of AD [3]. Although several genome-wide linkage screens for AD have been conducted, little is known about the relationship between genes modulating the extracellular matrix (ECM) and AD etiopathogenesis [4,5]. The ECM is a highly dynamic and heterogeneous network that plays a role in the regulation of tissue development, cell adhesion, and intercellular communication. Moreover, it is a reservoir of growth factors and cytokines. The most abundant proteins in the ECM are collagens. Several studies have shown that polymorphisms within genes encoding collagens can generate structurally and functionally abnormal connective tissues, which are more susceptible to mechanical stress, loss of epidermal integrity, and aging [6]. Furthermore, ECM collagens may play an important role in cutaneous immune responses due to the fact that they influence the migration of epidermal antigen-presenting Langerhans cells and T cells [7]. A clinical trial evaluating the effect of collagen tripeptide (CTP) on inflammation in AD revealed decreased inflammatory cytokines in keratinocytes after CTP treatment, and a reduction of the eruption area, SCORAD (scoring atopic dermatitis), and transepidermal water loss (TEWL) was observed [8]. Thus, genes encoding collagens may play a potential role in the pathogenesis of AD. Only a few studies have investigated some of these as potential biomarkers of AD susceptibility [9,10,11]. In our study, we have decided to investigate polymorphism in genes *COL3A1*, *COL6A5/29*, and *COL8A1*.

Our study aimed to estimate the associations between *Col3A1*/rs1800255, *Col6A5* /*29*rs12488457, and *Col8A1*/rs13081855 polymorphisms and the occurrence, course, and features of AD in the Polish population. The frequency of polymorphisms and their association with the severity of AD, pruritus, and coexisting asthma were examined.

## 2. Materials and Methods

### 2.1. Material

The study group included 157 AD patients (94 females and 63 males) from the Department of Dermatology, Venerology and Allergology, Medical University of Gdansk. The healthy controls consisted of 111 people (59 females and 52 males). The average age of AD patients was 20.0 years (SD =12.4, median age 18.0 years), and the average age of healthy controls was 30.0 years (SD = 11.7, median age 27.0 years). Among the AD patients, 49.2% have suffered from severe AD, 27.1% of patients from moderate AD, and the rest, 23.7%, from mild AD. Regarding the severity of pruritus in patients, 33.9% have suffered from mild pruritus, 27.1% from moderate pruritus and 30.5% from severe pruritus, and also 8.5% patients from very severe pruritus. A total of 11 patients have suffered from concomitant asthma. People who were subjected to immunosuppressive therapy or other immunotherapy (UV phototherapy, cyclosporine A, oral corticosteroid) such as breastfeeding and pregnant women, as well as people suffering from various cancers, inflammatory, or autoimmune diseases, were excluded from the study. The control group included people who had a negative individual and family history of atopic diseases, and a negative history of autoimmune diseases or neoplasms. Patients were classified as having AD according to the AD diagnosis criteria proposed by Hanifin and Rajka [12]. The study was conducted according to the guidelines of the Declaration of Helsinki and approved by the local ethics committee (no. 01-10023/0004978/01/253/0/2023). All participants provided signed informed consent. The clinical characteristics of AD patients and the individuals of the healthy controls are summarized in Table 1 and Table 2. The groups differed from each other in terms of age (*p* < 0.0001), whereas in terms of sex they did not (*p* = 0.3). Both the members of the study group and the healthy controls were Caucasian.

### 2.2. Methods

Whole blood samples were collected from test and control patients for genomic DNA extraction using Blood DNA Prep Plus according to the manufacturer’s protocols (A&A Biotechnology, Gdynia, Poland). Variant analysis of polymorphic genotypes for *Col3A1*/rs1800255, *Col6A5*/*29*rs12488457, and *Col8A1*/rs13081855 was performed using the polymerase chain reaction with sequence-specific primers (SSP-PCR). The severity of atopic dermatitis was assessed by the SCORAD scale and divided into the following categories: severe (SCORAD > 50), moderate (SCORAD 25–50), and mild (SCORAD < 25) [13]. Pruritus severity was estimated using a visual analogue scale (VAS) (<3 mild pruritus, 3–6.9 moderate pruritus, 7–8.9 severe, and 9–10 very severe pruritus) [14].

### 2.3. Statistical Analysis

The results of genotyping were statistically analyzed to evaluate the relationship with AD. Statistical calculations were made with Statistics, version 12.0 (StatSoft, Inc. 2015, Tulsa, OK, USA). Allele and genotype frequencies of analyzed SNPs were assessed by χ^2^ analysis. The Hardy–Weinberg equilibrium (HWE) was tested at each locus, and was used to test the significance of differences in the observed genotypes and alleles between the groups. The obtained data were included in the HWE for *p* > 0.05. Differences in median values between the groups were analyzed using the Mann–Whitney U test and the Kruskal–Wallis, while the correlation coefficients were assessed using Spearman’s rank correlation test. A logistic regression model was used to calculate the odds ratios (ORs) and the 95% confidence intervals (CIs).

## 3. Results

### 3.1. Genotyping for COL3A1 Gene Polymorphism rs1800255, COL6A5 Gene Polymorphism rs12488457, and COL8A1 Polymorphism rs13081855

We have estimated three genotypes for *COL3A1*/rs1800255 (AG, AA, GG) and *COL8A1*/rs13081855 (GT, GG, TT) in both groups. Regarding *Col6A5*/*29*rs12488457 polymorphism, two genotypes, AC and AA, were revealed. Biostatistical analysis of the polymorphisms of *COL3A1*/rs1800255, *COL6A5*/rs12488457, and *COL8A1*/rs13081855 did not reveal any significant differences in genotype distribution between AD and control subjects (*p* > 0.05) (Table 3).

### 3.2. The Association between COL3A1/ rs1800255 Polymorphism and SCORAD, Pruritus Severity, and Coexisting Asthma

**Figure 1:**
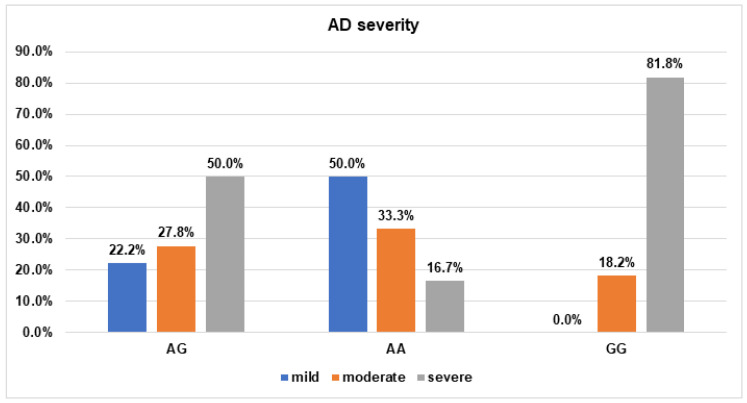
The frequency of AD severity occurrence measured by SCORAD for *COL3A1*/rs1800255 polymorphism. (Odds ratio AA (95% Cl): OR = 0.16 (0.03–0.79) *p* = 0.02; Odds ratio GG (95% Cl): OR = 6.3 (1.23–32.35) *p* = 0.03).

We have found that rs1800255 polymorphism in the *COL3A1* regarding the AA genotype was statistically associated with the occurrence of mild SCORAD (OR = 0.16; 95% Cl: 0.03–0.78; *p* = 0.02), while the GG genotype was significantly associated with severe AD measured by SCORAD (OR = 6.6 (95% Cl: 1.23–32.35, *p* = 0.03) (Figure 1).

**Figure 2:**
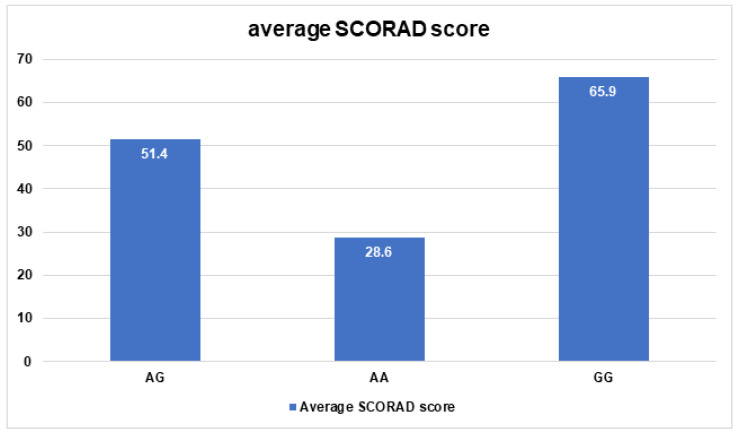
Average SCORAD scores for *COL3A1*/rs1800255 polymorphism.

Moreover, there was a significant relationship between *Col3A1*/rs1800255 polymorphism and the average SCORAD score (*p* = 0.008). Regarding this, patients with the AA genotype of *Col3A1*/rs1800255 significantly differed from patients with AG and GG genotypes (28.6 vs. 51.4 *p* = 0.01; 28.6 vs. 65.9 *p* = 0.0007, respectively). The group of patients with the GG genotype significantly differed only from patients with the AA genotype according to the average SCORAD score (65.9 vs. 28.6 *p* = 0.007). No significant differences were observed between GG and AG genotypes and AD severity (65.9 vs. 51.4 *p* = 0.3) (Figure 2).

**Figure 3:**
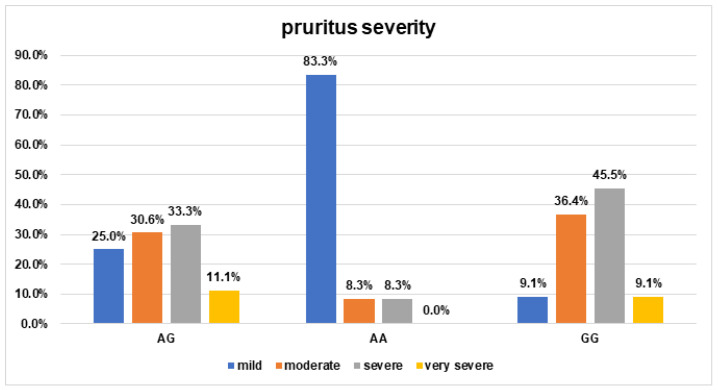
The frequency of pruritus severity occurrence in AD patients for *COL3A1*/rs1800255 polymorphism. (Odds ratio (95% Cl) for the AA genotype: OR = 18.5 (3.48–98.40) *p* = 0.0006).

Furthermore, the AA genotype of /*COL3A1*/rs1800255 was dominant in the group of patients with mild pruritus and was significantly associated with the occurrence of mild pruritus (OR = 18.5; 95% Cl: 3.48–98.40; *p* = 0.0006) (Figure 3).

**Figure 4:**
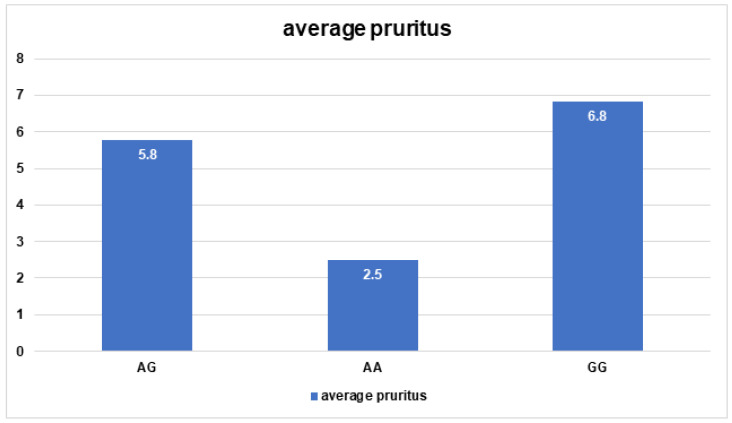
Average pruritus results for *COL3A1*/rs1800255 polymorphism.

In addition, according to the average pruritus level, the patients with the AA genotype of *Col3A1*/rs1800255 significantly differed from patients with AG and GG genotypes (2.5 vs. 5.8 *p* = 0.009; 2.5 vs. 6.8 *p* = 0.006, respectively). No significant differences were observed between GG and AG genotypes (6.8 vs. 5.8 *p* = 1.0) (Figure 4). No association between the presence of the SNPs of the *COL3A1* gene and concomitant asthma was found (*p* = 0.49).

### 3.3. The Association between COL6A5/29rs12488457 Polymorphism and SCORAD, Pruritus Severity, and Coexisting Asthma

**Figure 5:**
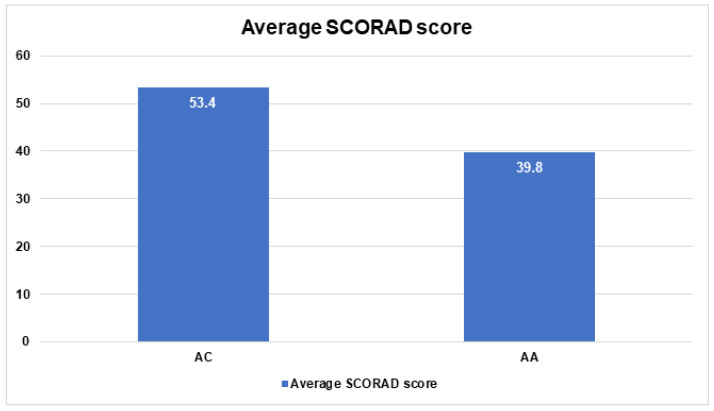
Average SCORAD scores for *Col6A5*/*29*rs12488457 polymorphism.

The average SCORAD score was significantly lower in the group of patients with genotype AA of *Col6A5*/*29*rs12488457 than the group of patients with the AC genotype of *Col6A5/29*rs12488457 (39.8 vs. 53.4; *p* = 0.04) (Figure 5). However, both average SCORAD scores are high, and correspond to moderate and severe courses of disease, respectively.

Regarding pruritus severity, no significant relationship between the frequency of occurrence of mild, moderate, severe, and very severe pruritus (*p* = 0.80) or average pruritus results and *Col6A5*/*29*rs12488457 polymorphism was revealed (*p* = 0.32). In addition, no association was found between *Col6A5/29*rs12488457 polymorphism and concomitant asthma (*p* = 0.60).

### 3.4. The Association between COL8A1/rs13081855 Polymorphism and SCORAD, Pruritus Severity, and Coexisting Asthma

No significant relationship between *Col8A1*/rs13081855 polymorphism and the AD intensification severity measured by SCORAD: mild, moderate, and severe (*p* = 0.15) nor average SCORAD score was found (*p* = 0.11). There was also no link between the frequency of occurrence of mild, moderate, severe, and very severe pruritus (*p* = 0.58) or average pruritus results and *Col8A1*/rs13081855 polymorphism (*p* = 0.29). In addition, no association was found between *Col8A1*/rs13081855 polymorphism and concomitant asthma (*p* = 0.87).

## 4. Discussion

Collagens constitute the main structural element of the ECM and provide tensile strength, regulate cell adhesion, support chemotaxis and migration, and direct tissue development [15]. The ECM as a highly dynamic network is constantly remodeling, and ECM components are deposited, degraded, or remodeled. This process plays an important role in development, wound healing, and normal organ homeostasis [16]. Immune cells as a regulator of remodeling ECM in AD have been reported [17]. Concerning genetic aspects of *COL* there are at present only a few studies discussing this topic, although the genetic background of the disease is well established. In our study, we have investigated the association of SNPs in three collagen-coding genes, *Col3A1*/rs1800255, *Col6A5/29*rs12488457, and *Col8A1*/rs13081855, with the clinical manifestations of AD as well as the coexistence of asthma. Significant results were obtained only in the cases of *COL3A1*/rs1800255 and *Col6A5/29*rs12488457 polymorphisms.

In humans, collagen type III is encoded by the *COL3A1* gene and is made up of only one collagen α chain [18]. The fact that it is secreted by fibroblasts and other mesenchymal cell types makes it a major player in various inflammation-associated pathologies [18]. It has been reported that the nerve growth factor (NGF) induces the production of type III collagen in chronic allergic airway inflammation [19]. In AD skin, the expression of the NGF is increased and associated with aggravation of the disease, which also indicates the possible involvement of collagen type III in AD pathogenesis [20]. Moreover, collagen type III is an important signaling molecule in the process of wound healing. It is synthesized as the first of all types of collagen in the early stages of wound healing and granulation formation [21]. The participation of collagen III in wound healing might be the potential explanation for the association between polymorphism in the *COL3A1* gene and the severity of AD revealed in our study in light of the remodeling process. We have observed that patients with the GG genotype of *COL3A1* presented more severe clinical symptoms of AD than patients with the AA genotype. Furthermore, in chronic lesions of AD, skin fibrotic remodeling is observed, corresponding to lichenification [22]. Fibrosis is considered to be the result of abnormal repair in response to chronic tissue damage caused by many factors, also including allergic responses. Chronic inflammation characterized by the upregulation of various proinflammatory cytokines and chemokines leads to the activation of fibroblasts and, consequently, the production of the ECM [23]. For fibrosis, the participation of type III collagen which is, in addition to type I collagen, a major component of ECM, is underlying [18]. Thus, various genetic variants of collagen III may influence its activity and deposition in tissue, and thereby be associated with clinical AD symptoms. Tissue remodeling in due course to the chronic inflammatory process associated predominantly with Th2 cytokines has been well established in asthmatic airways [24]. In AD, which is also associated with the Th2-type immune response, the process of tissue remodeling is not yet well understood. However, as mentioned above, it is known that chronic AD skin lesions undergo fibrotic remodeling. There is an observed increased collagen deposition and fibrocytes in human AD skin. It has been shown that TSLP is involved in promoting skin fibrogenesis in AD induced by IL-13 [22]. Another reported cytokine also described in the remodeling of asthmatic airways involved in the process of tissue remodeling in AD is IL-17. In the same study, the presence of remodeling markers, including procollagen-3, has been observed in AD skin lesions [25].

Considering our results, we may hypothesize that the remodeling process in AD is disturbed not only by proinflammatory cytokine storms, but also by a genetic condition. In light of the latest research from 2022 on the genesis of the atopic march, there were also indications for the hypothesis of ECM remodeling as a potential factor of disease progression in people affected by AD, not only by pro-inflammatory factors, but also by genetic background. The theory of the existence of the skin–lung axis as a communication method between tissues was presented on the example of other communication axes, such as the intestines and lungs, which could contribute to the propulsion of the atopic march and, further, skin remolding in AD by changing ECM gene transcription caused by pro-inflammatory factors. This study proved that inflammatory mediators caused the fibroblasts of AD patients to provide different gene transcripts, compared to control fibroblasts [26]. Therefore, the questions arise: what, then, triggers the remodeling of the ECM, and will finding this factor allows us to avoid the transition from the acute phase to the chronic phase and the progression of the disease? It is speculated that these may be distinct pro-inflammatory subsets of fibroblasts recently identified in AD [27]. However, the answers to these questions require further research. Perhaps considering this relationship in the context of AD itself is too narrow, and other diseases such as inflammatory bowel disease should be taken into account; it may be possible to find an answer that indicates one common factor for the above conditions, and reveal a different phenotype depending on individual susceptibility. This is just our hypothesis for now.

Polymorphism *COL3A1*/rs1800255 has been also linked with strokes in Chinese populations. The A allele of this gene appeared to be significantly associated with a reduced risk of lacunar stroke recurrence, while among atherothrombotic stroke patients it was significantly linked to an increased risk of all-cause death. On the other hand, the G allele increased the risk of stroke recurrence in patients with atherothrombotic stroke [28]. AD is considered a disease that has a relationship with the occurrence of stroke [29]. Our results suggest that the variant GG genotype of *COL3A1*/rs1800255 is associated with a severe course of AD, and as has been reported, the risk of stroke is significant in severe AD [29]. Thus, this polymorphism of *COL3A1*/rs1800255 may be a potential explanation for coexisting severe AD with stroke. However, it is only a hypothesis, because we did not directly investigate this association in our study. Importantly, the relationship between *COL3A1*/rs1800255 polymorphism and stroke was examined in the Chinese population. Thus, this group of patients was ethnically different from our population.

In human skin, the mRNA expression of *COL6A5,* previously marked with the symbol *COL29A1*, is present in the papillary dermis within the dermo-epidermal junctions and around some vessels of the reticular dermis [30]. The collagen type VI alpha 5 chain encoded by *COL6A5* is considered to play an important role in keratinocyte cohesion [11]. The human *COL6A5* gene is located on chromosome 3q22.1 nearness of the 3q21 locus, which was identified as an AD susceptibility region [31]. Early reports indicated SNPs in the *COL6A5* gene to be associated with susceptibility to AD [11]. Next, a pooling-based genome-wide analysis identified that the *COL6A5* gene was associated with atopy but was not consistently replicated [32]. The results from the comprehensive analysis of the *COL6A5* gene, also including the rs12488457 variant tested in our study, did not confirm the *COL6A5* SNP’s relationship with AD [9]. Strafella et al. analyzed Mediterranean populations consisting of patients with AD from Italy, Egypt, and Greece. They found no significant association, except for the Greek group. However, further analysis taking into account separate Greek cohorts and control subjects did not confirm this result [10]. In our population regarding *COL6A5*/29rs12488457 polymorphism, genotype distribution did not differ between AD and control subjects, which confirms previous reports about the lack of relationship between this gene’s polymorphism and AD susceptibility. However, the results have shown that according to the average SCORAD score, the patients with different genotypes differ from each other. The group of patients with the AA genotype of *COL6A5* presented significantly lower scores of SCORAD than patients with the AC genotype (39.8 vs. 53.4, *p* = 0.04). This may indicate that various polymorphic variants of this gene may influence the clinical course of AD. However, this proposal may have limitations. Of note is the fact that both average SCORAD scores, 39.8 and 53.4, are high, and correspond to moderate and severe courses of disease, respectively. The SCORAD score in many patients with a moderate to severe course of AD waxes and wanes between 39 and 55. Regarding this, the significance of specific genotype COL6A5-AA would be weakened in the correlation of clinical severity, although these scores can depend on when the assessment is conducted. In our study, the severity of AD in all patients was assessed before the treatment, which may influence the high SCORAD score of these patients.

Taking into account that collagen encoded by *COL6A5* is expressed in the epidermis and plays a role in keratinocyte cohesion, its involvement in the clinical course of AD [11] can be explained. The altered biological function of the collagen type VI alpha 5 chain may contribute to the loss of barrier integrity and enhance allergen penetration leading to the initiation of an inflammatory immune cascade, thereby influencing AD severity. It is worth adding that the above-mentioned studies did not investigate the relationship between polymorphism within the *COL6A5* gene and the clinical symptoms of AD, but only susceptibility to AD.

When it comes to polymorphism in the *COL8A1* gene, we observed no significant differences between AD and control subjects regarding the SNP *COL8A1*/ rs13081855 in our populations. Among Mediterranean populations, a significant association of *COL8A1*/rs13081855 (G/T) with susceptibility to AD has been found in Egyptian and Italian cohorts. The Greek cohort did not reveal this association [10]. These results may suggest ethnic differences. The authors of the above-mentioned paper also concluded that the polymorphism in the *COL8A1* gene may be a population-specific biomarker for AD [10].

We also investigated the effect of collagen gene polymorphism on the coexistence of asthma in patients with AD. The common feature of asthmatic airway remodeling is a thickening of the basement membrane resulting from the increased deposition of extracellular matrix (ECM) molecules, including structural proteins collagens I, III, and V [24]. Reeves et al. showed that *COL3A1* expression by human lung fibroblasts co-cultured with asthmatic airway epithelial cells were greater than in the control [33]. In our studied group, SNPs in any of the three studied collagen genes did not affect the occurrence of asthma. However, this may be a result of a very limited number of asthma patients participating in this study.

The major limitation of our study is the fact that the population was ethnically homogeneous, including only Caucasian people. In addition, this study included a limited number of patients, and the majority of patients presented a severe course of AD, which may influence results. Another limitation is that the number of patients with particular genotypes of collagens genes diversified. The study group and healthy controls were not age-matched, which is also a limitation of this study.

## 5. Conclusions

Our research suggests that genes modulating the ECM may also play a role in AD pathogenesis, providing new insights into AD pathophysiology. Specific genetic variants of *COL3A1* and *COL6A5* appear to be associated with the clinical course of the disease. The AA genotype of *COL3A1*/rs1800255 appears to be associated with the mild course of AD, and mild pruritus occurrence with the GG genotype appears to be associated with the severe AD course. Regarding *Col6A5*/*29*rs12488457, patients with the AA genotype present lower average SCORAD scores than patients with the AC genotype. At this stage, we would modestly suggest considering the polymorphisms of these particular genes encoding studied collagens as being a population-specific biomarker for the AD course. However, this requires further and broader studies, especially in terms of processes related to ECM remodeling, inter-tissue communication, and linking other autoimmune diseases in order to search for a common trigger. The modulation of collagens which are the primary component of the ECM may also serve as a therapeutic target of AD in new technologies.

## Figures and Tables

**Table 1:** The comparison of the age of the AD patients and the individuals of the healthy controls.

Group	Age						
	Average	SD	Minimum	Maximum	Q25	Median	Q75
AD patients *n* = 157	20.0	12.4	2.0	54.0	11.0	18.0	25.0
Healthy controls *n* = 111	30.0	11.7	6.0	61.0	23.0	27.0	35.0
*p*-value	<0.0001						

Abbreviations: AD: atopic dermatitis. In terms of age, groups differ statistically significantly (*p* < 0.0001).

**Table 2:** The comparison of the sex of the AD patients and the individuals of the healthy controls.

Sex	AD Patients *n* = 157 *n* (%)	Healthy Controls *n* = 111 *n* (%)	*p*-Value
Female	94 (59.8%)	59 (53.2%)	0.30
Male	63 (40.1%)	52 (46.9%)	

Abbreviations: AD = atopic dermatitis. In terms of sex, groups did not differ statistically significantly (*p* = 0.3).

**Table 3:** Distribution of *COL3A1*/rs1800255, *COL6A5*/*29*rs12488457, and *COL8A1*/rs13081855 genotype frequencies in AD patients and healthy controls.

Gene Polymorphism and Genotypes	AD Patients *n* = 157 *n* (%)	Healthy Controls *n* = 111 *n* (%)	* *p*-Value
*Col3A1*/rs1800255			
AG	97 (61.8%)	64 (57.7%)	0.30
AA	40 (25.5%)	37 (33.3%)	
GG	20 (12.7%)	10 (9.0%)	
*Col6A5*/*29*rs12488457			
AC	107 (68.2%)	67 (60.4%)	0.19
AA	50 (31.8%)	44 (39.6%)	
*Col8A1*/rs13081855			
GT	114 (72.6%)	84 (75.7%)	0.11
GG	37 (23.6%)	27 (24.3%)	
TT	6 (3.8%)	0 (0.00%)	

Abbreviations: AD: atopic dermatitis. The table presents the distribution of the genotypes for *COL3A1*, *COL6A5/29*, and *COL8A1* in patients and control groups. The distribution frequency of gene polymorphisms did not show any statistical significance. * Pearson’s chi-square test was applied.

## Data Availability

The data presented in this study are available in the PubMed database—https://pubmed.ncbi.nlm.nih.gov/ (accessed on 1 January 2023) or under the links cited of cited websites.
